# DRG Neurons Promote Perineural Invasion of Endometrial Cancer via GluR2

**DOI:** 10.7150/jca.40055

**Published:** 2020-02-10

**Authors:** Ting Ni, Ting Huang, Sheng-Lan Gu, Jing Wang, Yao Liu, Xiao Sun, Yu-dong Wang

**Affiliations:** 1 Department of Gynecology, International Peace Maternity and Child Health Hospital, Shanghai Jiao Tong University School of Medicine, No. 910 Hengshan Road, Shanghai 200030, China.; 2 Laboratory for Gynecologic Oncology, International Peace Maternity and Child Health Hospital, Shanghai Jiao Tong University School of Medicine, No. 910 Hengshan Road, Shanghai 200030, China.; 3 Shanghai Key Laboratory of Embryo Original Disease, Shanghai, China; 4 Shanghai Municipal Key Clinical Specialty, Shanghai, China

**Keywords:** endometrial cancer, GluR2, DRG neurons, perineural invasion, metastasis

## Abstract

**Background**: Perineural invasion (PNI) is correlated with negative prognosis in multiple cancers, but its role in endometrial cancer (EC) is still largely unknown; thus, targeted treatment for nerve infiltration is lacking as well.

**Methods**: The interaction between nerve and EC cells were investigated by in vitro neural invasion assay and transwell coculture system. Then the nerve-related receptor gene glutamate ionotropic receptor AMPA type subunit 2 (GRIA2) was detected in EC tissues and cells using PCR array, western blotting, and immunohistochemistry. The role of GluR2 (gene name GRIA2) on EC proliferation, migration and invasion was evaluated by a GluR2 antagonist and shRNA. At the same time, the neurotransmitter effect on GluR2 (glutamate) from the cocultured conditional medium was measured using high-performance liquid chromatography (HPLC).

**Results**: EC cell line Ishikawa (ISK) showed the ability to migrate along neurites in vitro and the numbers of migrated/invaded EC cells in the DRG neuron coculture group were significantly increased. The expression of GluR2 in EC tissue was found to be higher than that in para-carcinoma tissue. After GluR2 antagonist and GluR2 shRNA treatment, the proliferation, migration and invasion of ISK cells was markedly inhibited. Moreover, the ability of DRG neurons to promote the migration and invasion of ISK cells could also be attenuated by downregulation of GluR2, and the concentration of the neurotransmitter glutamate was notably increased in the coculture conditional medium compared to that in the DRG neuron or ISK cells alone.

**Conclusions**: DRG neurons promote metastasis of EC cells via GluR2, which might be a risk factor for PNI in EC. Moreover, the perineural system may promote tumor invasion and metastasis under certain circumstances.

## Introduction

Endometrial cancer (EC) is one of the most common gynecological malignancies threatening women worldwide with 61,380 estimated new cases and 10,920 deaths in the United States in 2017 1. Distant metastasis is still the leading cause of death in EC 2. Despite the progress in surgical and conservative treatment for EC, patients with tumors spread beyond the uterus might progress within 1 year 3-4. In addition to direct invasion and lymphatic and hematogenic spread, perineural invasion (PNI) is a fourth route of tumor dissemination leading to poor prognosis in multiple cancers, yet the mechanisms are still largely unknown 5-7. According to recent literature, denervation could suppress gastric tumorigenesis and concurrently enhance the chemotherapy effect 8. Currently, it is unclear whether PNI is a high-risk factor of poor outcome in EC patients, and it is also urgent to demonstrate the interaction between tumor cells and nerves in EC.

PNI has been defined as the infiltration of the perineural sheath by tumor cells 9-10 and plays a crucial role in the progression of several carcinomas, including head and neck squamous cell carcinoma 11, pancreatic cancer 12 and prostate cancer 13. Biological interactions between nerves and cancer cells are vital in the development of PNI. The abnormal release of neurotrophic factors, neurotransmitter and neuropeptide was found to be correlated with PNI in several cancers 11-12,14. However, the key drivers of PNI in cancers have still not been identified. Glutamate as one of the essential neurotransmitters in the nervous system also regulates the growth of cancers 15-16. A study by Rzeski et al. demonstrated that glutamate antagonist could decrease motility and invasion of cancer cells 17. Since the blockage of α-amino-3-hydroxy-5-methyl-4-isoxazolepropionic acid receptors (AMPARs) could suppress migration and promote apoptosis in cancers 18, AMPARs might play an important role in cancer metastasis, especially in PNI. Interestingly, the role of GluR2 (a subtype of AMPARs) in cancer biology is conflicting. It is reported that GluR2 plays the opposite effect on tumor progression in glioma cells 19 and hepatocellular carcinoma 20. Moreover, the effect of GluR2 is even diversified in different cell lines of the same cancer 21. Thus, exploring the role of neurotransmitter/neuropeptide and its receptors in PNI of EC, especially GluR2, is both challenging and emergent.

Endometrial nerve fibers play an important role in endometriosis, adenomyosis and uterine fibroids 22-23. However, little is known of PNI in EC as well as the interaction between EC cells and nerves. In the present study, we set out to investigate the effect of nerves on tumor proliferation and metastasis with an emphasis on nerve-related receptors.

## Materials and Methods

### Cell culture

Human EC cell lines (KLE, HEC-1A and Ishikawa) were obtained from the American Type Culture Collection (ATCC, Manassas, VA, USA). Cell lines were authenticated using Short Tandem Repeat (STR) analysis as described in 2012 in ANSI Standard (ASN-0002) by the ATCC Standards Development Organization (SDO) in January and February, 2018. Other cell lines including cervical cancer cell line Hela and uterine leiomyoma cells SK-UT-1 were purchased from the Cell Bank of the Chinese Academy of Sciences (Shanghai, China). Cells were cultured in Dulbecco's modified Eagle medium (DMEM)-F12 (Gibco, Auckland, New Zealand) supplemented with 10% fetal bovine serum (Gibco, Carlsbad, CA, USA), 100 μg/ml of penicillin and 100 U/ml of streptomycin (Gibco) in a humidified atmosphere of 5% CO_2_ at 37°C.

DRGs were dissociated from the female Sprague Dawley rats (body weight 100-150 g). DRG neurons were further dissociated according to a previous study by Su et al. 24. Freshly dissected DRGs were digested in the mix of DMEM containing 1 mg/ml of collagenase type 1A, 0.4 mg/ml of trypsin type I, and 0.1 mg/ml of DNase I (all from Sigma) at 37°C for 20-35 minutes. Subsequently, the cells were plated on poly-D-lysine precoated 24-well plates in DMEM containing 10% fetal bovine serum (Gibco). After 4 h, the medium was replaced with DMEM/F12 (1:1; Gibco) containing 1% N2 (Invitrogen).

### In vitro neural invasion assay

The in vitro Matrigel/DRG model was set up by Huyett 25 and was frequently used to investigate PNI in several cancers. DRGs are harvested from the spinal column of a sacrificed Sprague Dawley rat and placed in the center of 2.5μl of matrix. Cancer cell lines were placed peripherally around the matrix 48 hours later. After 72 hours cocultivation, cancer cells were stained with Pan-cytokeratin to show their movement towards DRGs.

### Migration, invasion and coculture assays

For migration assays, a total of 1x10^5^ Ishikawa cells in 200μl of serum-free DMEM medium was seeded into the upper chamber of a 24-well polycarbonate transwell filter (8μm pore, Corning Inc., Glendale, AZ, USA), and 600μl of complete medium was added to the lower chamber. The cells were treated with GluR2 antagonists (10μm, PEP2M, TOCRIS, UK) and agonist (10μm, CI-HIBO, TOCRIS, UK). The cells were fixed with 4% paraformaldehyde, stained with crystal violet staining solution (Beyotime, Shanghai, China) and counted at 200x magnification in five random fields/well. The invasion of cells was performed using transwell chambers with 8μm pore membranes precoated with 50 μl of Matrigel at 1:6 dilution (BD Biosciences, San Jose CA, USA) on the upper side at 37°C for 1 h. The following process was the same as described above. As in the indirect coculture system using transwells, Ishikawa cells were seeded in the upper chamber, and DRG neuron cells were seeded in the lower chamber. Migrated cells were counted as previously described. In the meantime, the condition medium after coculture was collected and used in subsequent experiments.

### Wound-healing assay

Ishikawa cells were seeded in 6-well plates and allowed to reach a subconfluent state. The cell monolayer was scratched using a sterile 100μL pipette tip. Then, the cells were treated with serum-free medium containing 10μm GluR2 agonist and serum-free medium containing 10μm GluR2 antagonist. The wounds were photographed at 0, 24 and 48 h, and the cell migration distance was calculated from the photomicrographs (percentage of wound-healing: 0-24 h width of wound/0 h width of wound or 0-48 h width of wound/0 h width of wound).

### Clinical samples

Paraffin-embedded endometrium tissue samples without preoperative radiotherapy/chemotherapy were obtained from the International Peace Maternity and Child Health Hospital from 2013 to 2015. For the IHC test, 39 EC tissues were obtained from patients who underwent surgical staging according to the criteria of the Federation International of Gynecology and Obstetrics (FIGO) 2009 staging system. Fourteen normal endometrium samples were collected from patients with benign disease such as myoma, adenomyosis or other diseases that underwent hysterectomies. Thirteen endometrial atypical hyperplasia (AH) samples were collected from patients who underwent hysteroscopy due to abnormal vaginal bleeding. For PCR arrays, five more EC tissues and three paired para-carcinoma tissues were also collected. For immunofluorescence assay, nine normal tissues, 20 AH samples and 58 EC tissues were involved. The section of EC sample with PNI and without PNI was stained with haematoxylin & eosin (H&E). The ethics committee of the International Peace Maternity and Child Health Hospital approved the study, and written consent was obtained from all participants.

### Quantitative reverse-transcription PCR (qRT-PCR), western blotting and immunohistochemistry (IHC)

Total RNA extraction, qRT-PCR, western blotting and the IHC process were performed as described previously in our group 26-27. The primers used for qRT-PCR are as follows: GAPDH, forward primer 5′-ACAACTTTGGTATCGTGGAAGG-3′ and reverse primer 5′-GCCATCACGCCACAGTTTC-3′; GRIA2, forward primer 5′-CACCCCACA TCGACAATTTGG-3′ and reverse primer 5′-GACGTGGAGTGTTCCGCAA-3′; HTR3E, forward primer 5′-GGAAGGGGCGTTACTTTCACC-3′ and reverse primer 5′- CGGACGGAAGGGCTTTCTAT-3′; ADRA2B, forward primer 5′-AGAGGTCAACGGACACTCGAA-3′ and reverse primer 5′-CCCCACAAACACCCTCCTT-3′; NR4A1, forward primer 5′-ATGCCCTGTATCCAAGCCC-3′ and reverse primer 5′-GTGTAGCCGTCCATGAAGGT-3′; SSTR4, forward primer 5′-CTGTTGGTCACTCTCCCCAT-3′ and reverse primer 5′-GATTTTCTTCTCCGAGCGCC-3′; SSTR1, forward primer 5′-GCGCCATCCTGATCTCTTTCA-3′ and reverse primer 5′-AACGTGGAGGTGACTAGGAAG-3′. Monoclonal mouse antibody against GRIA2 antibodies used in western blotting was purchased from Abcam (AB106515, 1:2000, UK). For evaluation of GRIA2 IHC staining, two experienced pathologists blinded to patients' clinical pathological characteristics were involved. Staining intensity was scored as 0 (negative), 1 (weak), 2 (moderate) or 3 (strong). The percentage of positive cells was scored as 0 (0-5%), 1 (6-25%), 2 (26-50%), 3 (51-75%), and 4 (76-100%). And the samples with inconsistent scores by two pathologists were further discussed and decided. Finally, multiplying these two scores calculates immune reactivity scores (IRS) 28.

### Immunofluorescence assay

For immunofluorescence, the paraffin-embedded endometrium tissues were sliced into 4μm sections, dewaxed with xylene and rehydrated with graded alcohol. Ethylene diamine tetra-acetic acid (EDTA) was applied for antigen retrieval. Nonspecific binding was blocked with goat serum (BOSTER, AR1009, USA). Sections were incubated with antibody to NF-L (1:200, Cell Signaling Technology, 2837T, USA) and Pan-cytokeratin (1:400, Cell Signaling Technology, 4545T, USA) at 4℃ overnight followed by secondary antibody conjugated with Alexa Fluor® 488 (1:200; Thermo Fisher Scientific). Images were obtained by fluorescence microscopy (Leica, Germany). The positive nerve fibers were counted in 10 representative fields (20x10) without knowing the histological diagnoses and clinical data. The total number of fibers of each patient were added together and further analyzed. For identification of DRG neurons, cells were fixed with 4% paraformaldehyde (PFA) and then incubated with the primary antibody MAP2 (1:200, Cell Signaling Technology, 8707T, USA) at 4℃ over-night. After that, cells were incubated with secondary antibody conjugated with Alexa Fluor® 488 (1:200; Thermo Fisher Scientific). Cells were counterstained with DAPI (Thermo Fisher Scientific) and examined under a fluorescence microscope (Leica, Germany).

### Proliferation assay

Cell proliferation was detected by cell counting kit-8 (CCK8, Dojindo, Japan) according to the manufacturer's instructions. A total of 4000 cells per well were seeded in a 96-well plate in complete culture medium with or without GluR2 agonist or antagonist. The absorbance values were calculated at 450 nm using a Spectra Max 190 microplate reader (Bio-Rad Model 680, USA).

### Transfection with lentivirus

Lentivirus carrying small hairpin RNA (shRNA) targeting GRAI2 (hU6-MCS-Ubiquitin-EGFP-IRES-puromycin; Genechem, Shanghai, China) was transfected into cells at 70% confluency in 12-well culture plates. The primary medium containing 1% FBS opti-MEM (Invitrogen) was complement with 20% FBS medium after 6 h. Cells were incubated for another 72 h before harvest. Stable colonies were selected using 1 μg/mL of puromycin (Genechem, Shanghai, China) for one week. Knockdown of target gene was validated by qRT-PCR and western blotting.

### PCR array

EC tissues and paired para-carcinoma tissue of the same EC patients were applied for the PCR array test. The expression of 123 nerve-related receptors in both tumor and control group were detected by BioTNT (BioTNT Biotechnologies Co., Ltd. China) according to the manufacturer's instructions.

### Glutamate concentration measurement

The cocultured conditional medium of Ishikawa cells and DRG neurons for 24 h was collected. Perchloric acid was added to the medium with a final concentration of 5% and then centrifuged at 12,000 g for 20 min twice. DMEM, supernatant of Ishikawa cells or DRG neuron alone was also applied as certain controls. The concentration of glutamate was detected using HPLC.

### Statistical analysis

Results from triplicate experiments are presented as the means with standard deviations (SD). Data were analyzed using the SPSS 22.0 software (SPSS, Chicago, USA) and GraphPad Prism 6 Software (GraphPad Software). Two-tail unpaired Student's t-test or one-way analysis of variance (ANOVA) was involved in statistical analysis. P-value<0.05 was considered statistically significant.

## Results

### PNI in endometrial cancer

**Figure 1:**
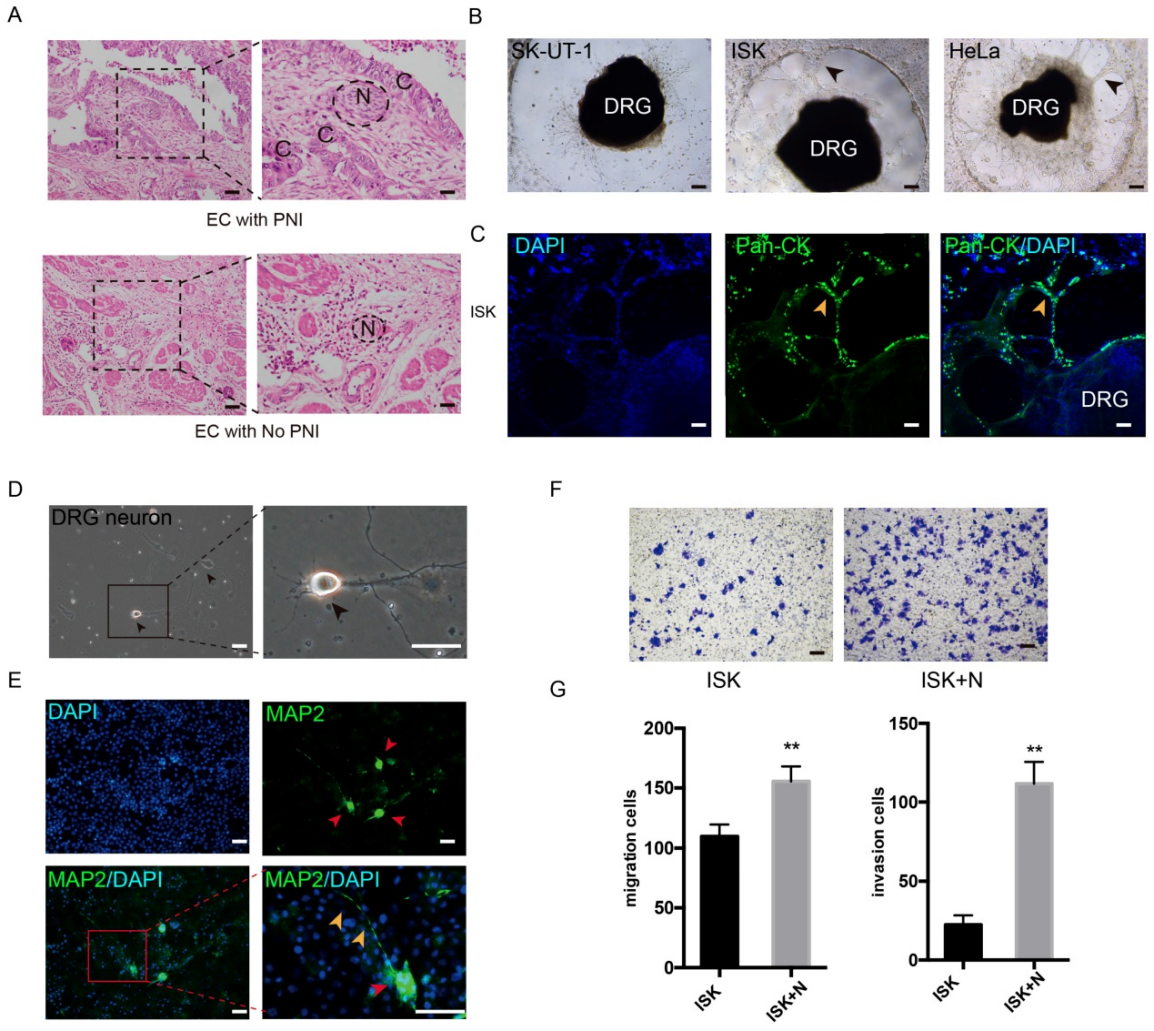
** DRG neurons promote the migration and invasion of EC cells.** (A) EC tissue with or without PNI was stained in Hematoxylin and eosin (HE). The nerve area was marked by dashed lines. N = nerves; C = cancer cells. (B-C) DRG was placed in the center of Matrigel. The interaction of EC cell line and DRG was confirmed in bright field (black arrow) and immunofluorescence staining (yellow arrow). ISK cells were stained by pan-cytokeratin (pan-CK), green, scale bar, 100 μm. (D) Representative bright field image of DRG neurons after culture for 3 days (black arrow); scale bar, 50μm. (E) Immunofluorescence staining of DRG neurons. The dissociated DRG neurons were defined as DAPI+/MAP2+ (red arrow: DRG neuron, yellow arrow: nerve fiber); scale bar, 50μm. (F) Transwell coculture systems were involved to evaluate the migration/invasion effect of DRG neurons on EC cells; scale bar, 100μm. (G) The migrated/invaded cells were then counted in five random fields and statistically analyzed. Data are presented as the mean±SD; **P<0.01.

The PNI phenomenon was confirmed in certain specimens of EC (Figure 1A). The potential of PNI in SK-UT-1, ISK, KLE, HEC-1A and Hela was separately assessed by cocultivation with DRGs. In addition to cervical cancer cell line Hela, the EC cell line ISK also showed notable ability to interact with DRG neurites, while HEC-1A and KLE demonstrated low interact with nerve cells (Figure 1B, Figure S1A). After 72 hours of cultivation, ISK cells marked with Pan-cytokeratin moved into the Matrigel and spread along the neurites, which was in a similar manner with Hela cells (Figure 1C, Figure S1B). These results identified the presence of PNI in EC.

### DRG neurons promote the migration and invasion of EC cells

To further determine the effect of nerves on EC cells, DRG neurons were dissociated from Sprague Dawley rats. DRG neurons were defined as DAPI+/MAP2+ (Figure 1D-E). The neurons were then mixed thoroughly and seeded in the lower well of the transwell indirect coculture system, and the EC cell line Ishikawa cells were seeded in the upper well for 24 h. The number of migrated and invaded cells was significantly increased in the coculture group compared to that in the control group (Figure 1F-G), which indicates that DRG neurons promote the metastatic ability of EC cells.

### Nerve-related receptor gene GRIA2 was highly expressed in EC tissue compared to that in para-tumor tissue

**Figure 2:**
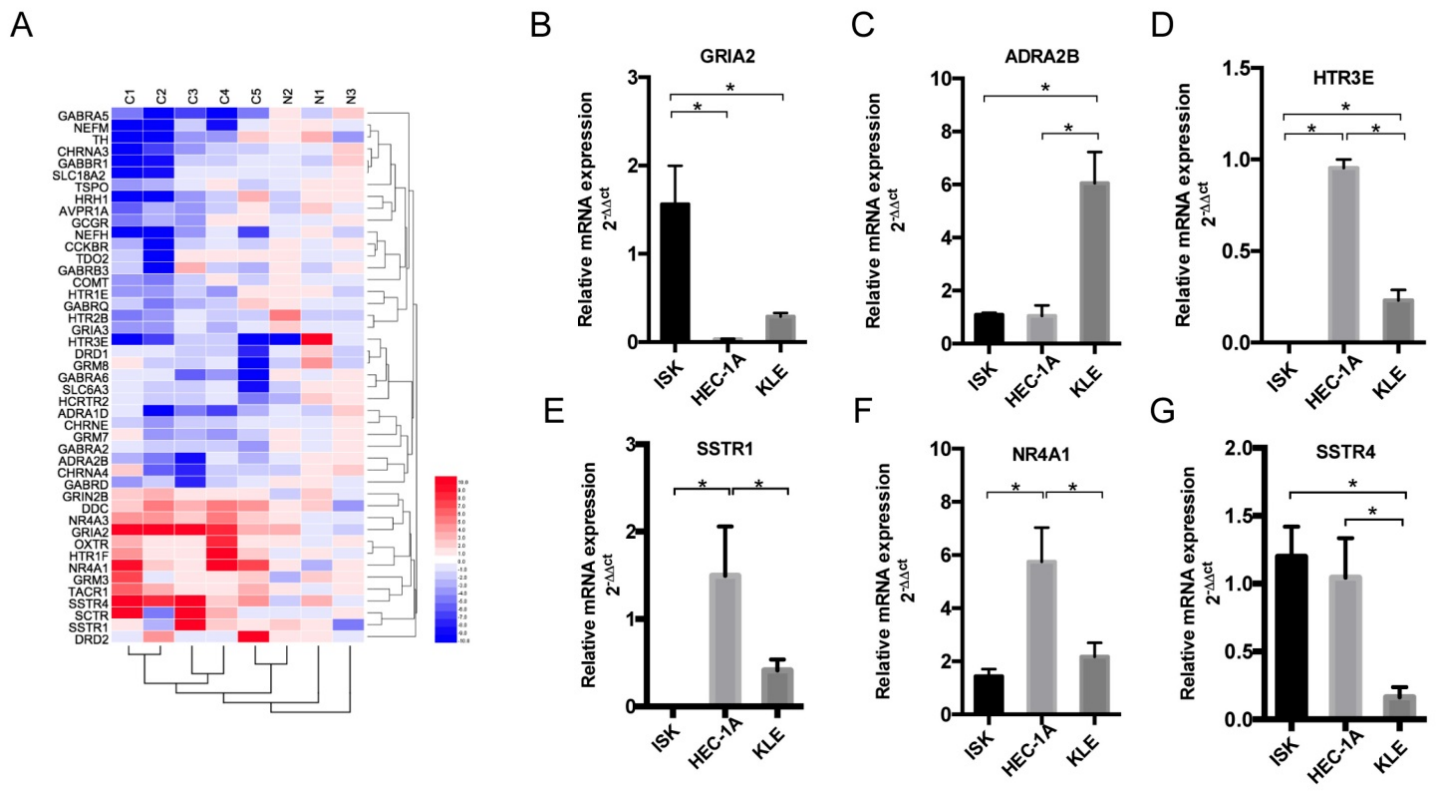
** Expression of nerve-related receptor genes in EC tissue and cell lines.** (A) Clustered heat map of differentially expressed nerve-related genes in endometrial cancer and para-cancer normal tissue (C1-C5 represents cancer tissues, N1-N3 represents normal tissues); 45 genes displayed a ≥2-fold increase or decrease in expression level. Each color patch represents the expression level of related genes with a continuum of expression levels from blue (lowest) to red (highest). (B-G) Expression of interested nerve-related receptor genes in EC cells lines (Ishikawa, HEC-1A and KLE) detected at the mRNA level; *P<0.05.

To detect the different expression levels of nerve-related receptors in EC and the paired control group, PCR array was involved in this study. Among the 123 nerve-related genes, the results revealed that the average GRIA2 expression was 10.58-fold higher in EC tissues than in the normal control group (Supplementary Table). Furthermore, 45 genes displayed a ≥2-fold increase or decrease in expression level were displayed in Figure 2A. Each color patch represents the expression level of related genes with a continuum of expression levels from blue (lowest) to red (highest), and the expression of interested nerve-related receptors including GRIA2, SSTR1, SSTR4, HTR3E, ADRA2B, and NR4A1 was also detected in EC cell lines at the mRNA level (Figure 2B-G). Highly expressed GRIA2 was also expressed in EC cell lines (especially in Ishikawa cells), indicating that it might play an important role in endometrial cancer and nerve crosstalk.

### GluR2 (encoded by the GRIA2 gene) is expressed in EC tissue and cells

**Figure 3:**
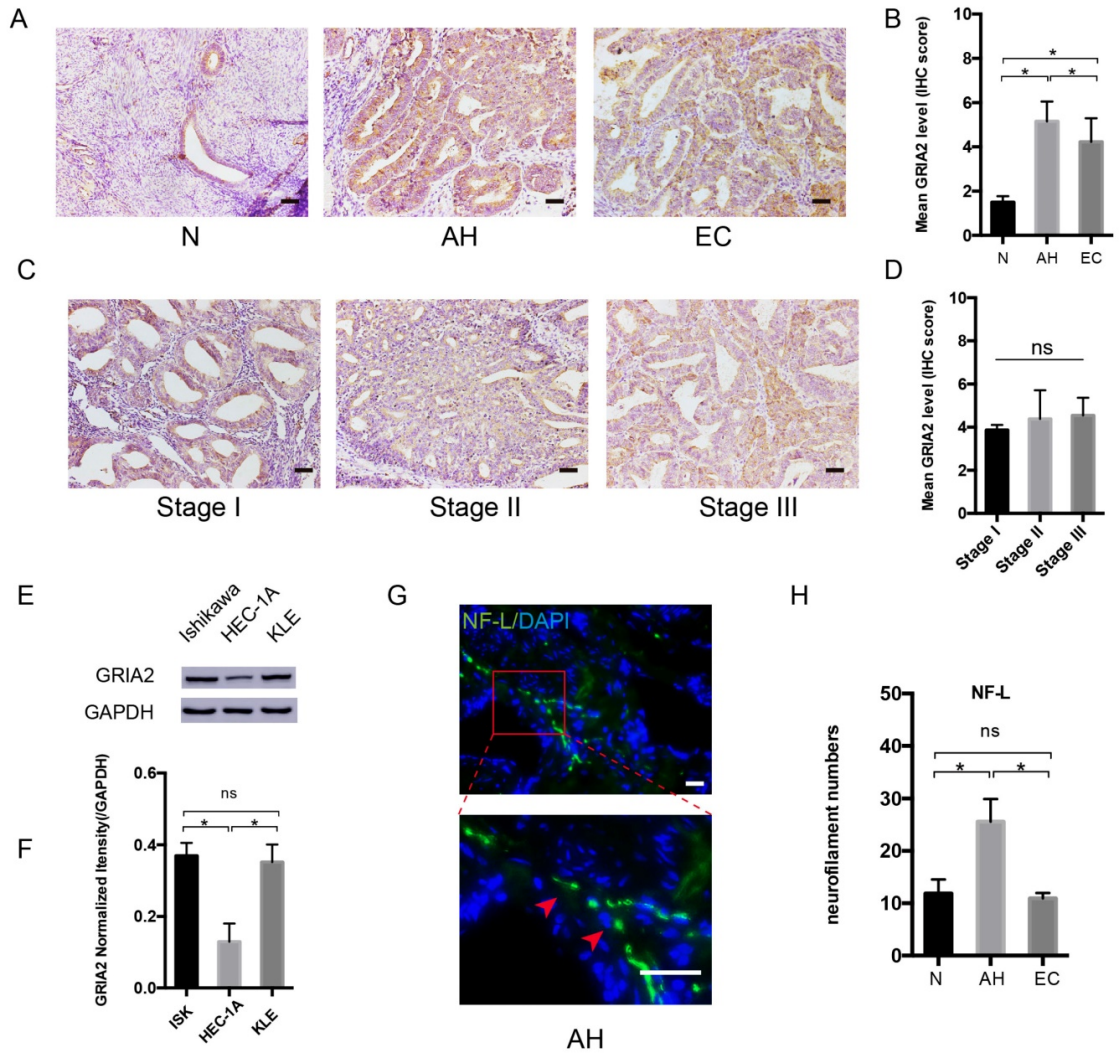
** GluR2 is expressed in EC cell lines and tissues.** (A-D) IHC analysis of GluR2 in normal endometrium (N), atypical hyperplasia endometrium (AH), EC and stage I-III EC tissue. The mean level of GluR2 (encoded by GRIA2 gene) was significantly higher in AH than in normal and EC groups, whereas no significant difference was detected in different EC stages. Semiquantitative analysis of IHC staining was practiced among the three groups; scale bar, 50μm. (E, F) The expression of GluR2 at the protein level in EC cell lines was detected by western blotting, and the results were further quantified by densitometry three times. (G) Representative image of neuro-filament light chain (NF-L) in AH tissues (the long and linear fiber stained in green, red arrow); scale bar, 50μm. (H) The number of nerve fibers in normal, AH and EC tissues were counted in 10 random fields/slide and statistically analyzed; scale bar, 50μm. Data are presented as the mean ± SD; *P< 0.05.

After PCR array, the highly expressed GRAI2 was further confirmed at the protein level in EC cell lines and EC tissue. The relative expression of GluR2 was higher in EC tissue than in the normal control group. Interestingly, GluR2 expression in atypical hyperplasia (AH) endometrium was much higher than those in the EC and normal groups (Figure 3A and B). As in different EC stage groups, the expression of GluR2 was not significantly changed (Figure 3C and D), and GluR2 was also detected in EC cell lines at the protein level; it was highly expressed in ISK and KLE cells compared to that in HEC-1A cells (Figure 3E-F). The neurofilament light chain (NF-L) was also expressed higher in AH endometrium than in EC and normal groups (Figure 3G-H). In this part, the results indicated that precancerous EC cells might already be able to initiate PNI. Meanwhile, the number of nerve fibers might also play an important role in PNI.

### GluR2 antagonist inhibits proliferation, migration and invasion of EC cells

**Figure 4:**
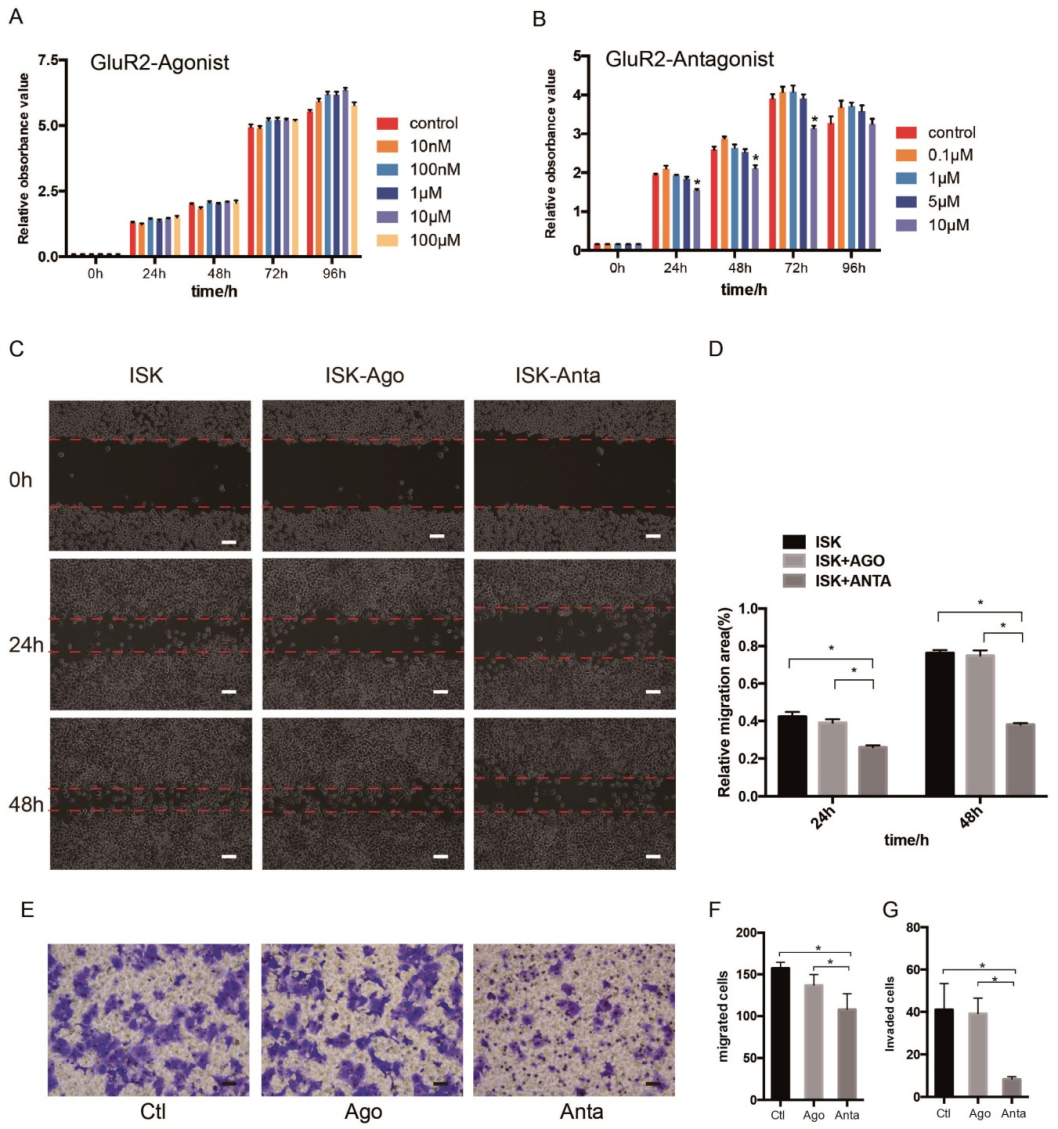
** GluR2 antagonist decreases proliferation, migration and invasion of EC cells.** (A-B) Ishikawa cells were treated with GluR2 agonist from 10nM to 100μM or antagonist from 0.1μM to 10μM for 0-96 h. CCK-8 assay was used to test the cell proliferation ability. (C-D) ISK cells treated with GluR2 agonist/antagonist (both at 10μM) and its control were subjected to wound-healing migration assay. Representative images of wounds at different time points (0, 24, 48 h) were displayed, and the percentage of wound closure was measured (percentage of wound healing: 0-24 h width of wound/0 h width of wound or 0-48 h width of wound/0 h width of wound). (E-G) Transwell migration/invasion assays were applied to evaluate the ISK cells while treated with GluR2 agonist or antagonist; scale bar, 50μm. Data are presented as the mean ± SD; *P< 0.05.

As the expression of GluR2 at both the mRNA and protein level was confirmed, we subsequently detected the proliferation, migration and invasion of EC cells when exposed to GluR2 agonist and antagonist using cell counting kit -8 (CCK-8) test. In our study, however, the GluR2 agonist has no effect on ISK cell viability. The GluR2 antagonist at 10 μm could significantly inhibit the cell viability of ISk (Figure 4A-B). To investigate the effect of GluR2 on migration of EC cells, both wound-healing and transwell migration assay were applied. The GluR2 agonist did not promote the migration ability of ISK, whereas the antagonist inhibited the migration ability (Figure 4C-F). The transwell assay for invasion revealed similar results to those of the migration assay (Figure 4G).

### Endogenous and exogenous knockdown of GluR2 decreases proliferation, migration and invasion of endometrial cancer cells

**Figure 5:**
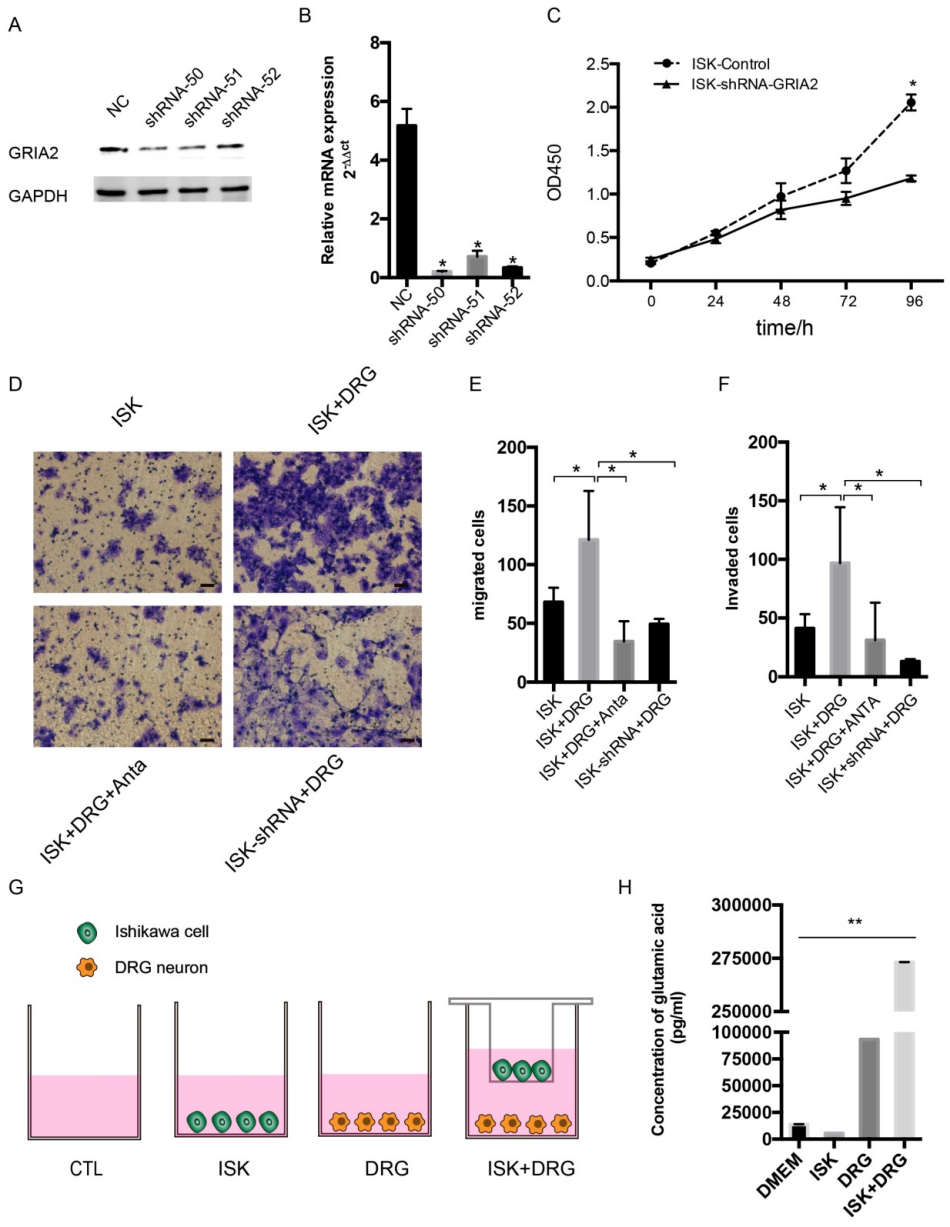
** Knockdown of GluR2 decreases proliferation, migration and invasion of endometrial cancer cells.** (A-B) Ishikawa cells were transfected with negative control shRNA or shRNA-GRIA2 (50, 51, 52). Downregulation of GRIA2 was verified in whole cell lysates from these cells at both the RNA and protein level, especially in the shRNA-50 group. (C) After infection with GRIA2 shRNA, stable colonies were selected using 1μg/mL of puromycin, and the proliferation rate of cells (both control and infected cells) were detected using the CCK-8 assay at different time points (0, 24, 48, 72, and 96 h). (D) Transwell assay was used to test the migration and invasion ability of EC cells (including control ISK cells, ISK+DRG neurons, ISK+DRG neurons+GluR2 antagonist at 10μM and ISK-shRNA + DRG neurons); scale bar, 50μm. (E- F) The migrated/invaded cells were counted in five random fields and statistically analyzed. Data are presented as the mean ± SD. (G- H) The concentration of glutamic acid in four different groups were tested by HPLC; *P < 0.05, **P < 0.01.

To determine the function of GluR2 in EC cells, shRNA targeting GluR2 was transfected into Ishikawa cells. The knockdown effect of shRNA was validated by western blotting and qPCR, and shRNA-50 is the most effective (Figure 5A-B). Thus, it was used in the subsequent assay. The proliferation rate of ISK cells was inhibited after infection with GRIA2 shRNA (Figure 5C). Both endogenous and exogenous knockdown of GluR2 decreases the migration and invasion ability of endometrial cancer cells as well (Figure 5E-F). Interestingly, the amounts of secreted glutamate in Ishikawa cell supernatants were much lower than that in the DRG neuron coculture group detected by HPLC assay. The level of glutamate was 273180.8±10.8 pg/ml in the coculture conditional medium, 5516.1±8.7 pg/ml in the DRG neuron, 93160.8±9.4 pg/ml in Ishikawa cells and 13857.3±68.6 pg/ml in DMEM alone.

## Discussion

**Figure 6:**
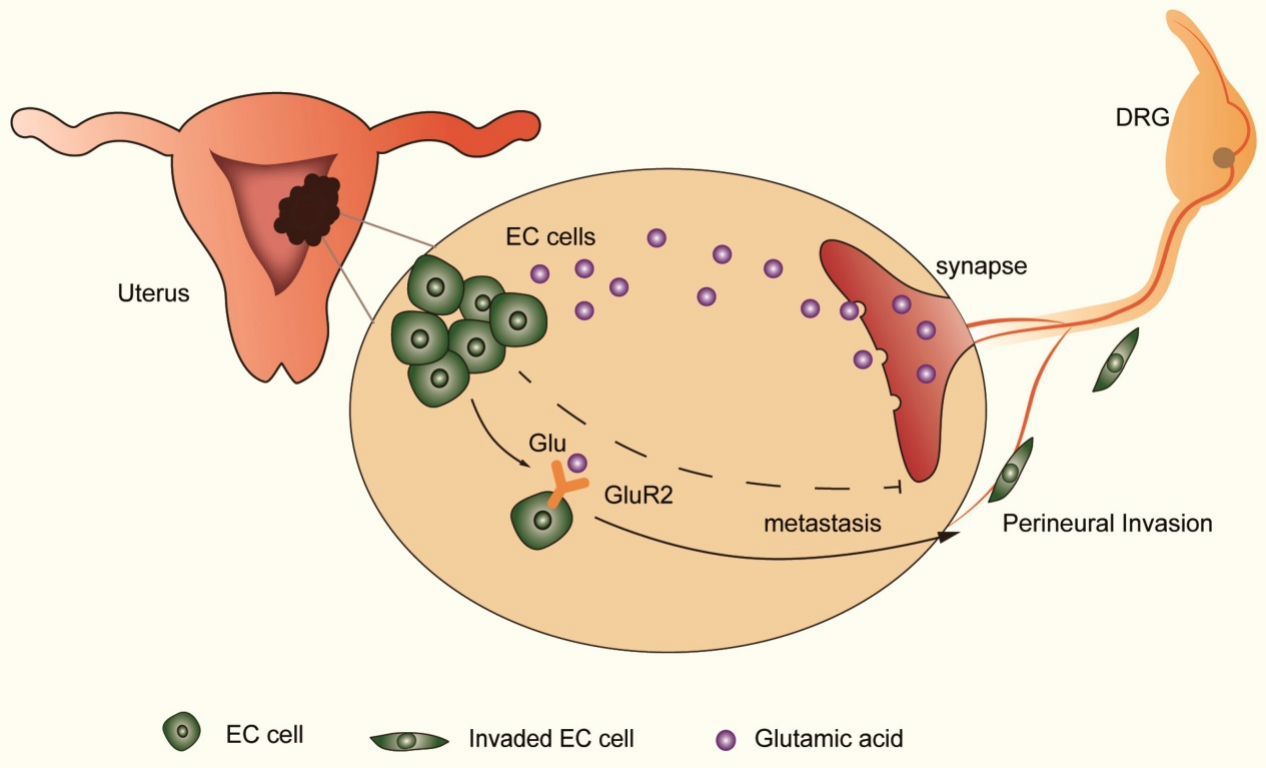
** DRG neurons promote perineural invasion (PNI) of EC cells via GluR2.** DRG neurons could release more glutamic acid when cocultured with EC cells. Then, the high-concentration glutamic acid activates tumor-expressed GluR2. The GluR2 activation promotes the migration and invasion of EC cells and contributes to PNI in EC as well. Although both endogenous and exogenous knockdown of GluR2 inhibited the ability of metastasis in EC cells, targeting GluR2 might block PNI by disrupting nerve-cancer crosstalk.

The distribution of neural and neurotransmitter signals as a part of the tumor microenvironment is important in cancer progression 29-31. Understanding the effect of nerve and targeting the regulatory mechanism is important in improving cancer survival. Using the transwell coculture system and focusing on nerve-tumor interactions in EC, our results showed that DRG neurons facilitate the migration and invasion of EC cells by activating GluR2 (Figure 6). In this process, elevated glutamate plays an important role. Moreover, both endogenous and exogenous knockdown of GluR2 can inhibit the migration and invasion of EC cells. Thus, our findings demonstrate the interaction between nerves and EC cells, providing a potential target to interrupt the nerve-cancer crosstalk.

Recent studies have revealed that nerves could infiltrate the tumor microenvironment and stimulate the growth and dissemination of cancer cells 5, 32-33. Nerve fibers were reported to play an important role in the endometrium 22-23; However, whether nerves facilitate the progression of EC is still unknown. In our study, DRG neurons promote the migration and invasion of EC cells. The expression of nerve-related receptors in tumor and para-tumor tissues was also diverse. The results indicated that nerves play a crucial role in the metastasis of EC cells, and the interaction between nerve and cancer cells might be a potential mechanism of PNI in EC.

Stage II endometrial cancer patients with gross cervical involvement may be treated with radical hysterectomy 34. However, the radical surgery might disrupt the autonomic nervous system in the pelvis and result in sexual or voiding dysfunction 35-36. Nerve-sparing radical hysterectomy (NSRH) was recommended by some studies as it improved the sexual function/quality of life in cervical and endometrial cancer 37-40. As PNI was significantly associated with poor prognosis in several cancers, the outcome of practicing NSRH remains unclear. According to our study, the expression of neuro-filament light chain (NF-L) was higher in AH than in EC and normal endometrium. The result suggested that nerve fibers might grow toward precancerous cells in early carcinogenesis. One study in prostate cancer revealed that the density of nerve fibers in tumor or para-tumor tissue was associated with poor clinical outcomes 13. Thus, the distribution of nerve fibers likely predicts PNI and guides the clinical practice of NSRH.

AMPA receptors have been shown to promote tumor progression in several cancers including glioblastoma 41, glioma 19,42 and hepatocellular carcinoma 20. In the case of Ca^2+^-impermeable AMPA receptor GluR2, its role in cancer biology is conflicting. In pancreatic cancer, RNAi of AMPA receptors (both Ca^2+^-impermeable GRIA2 and Ca^2+-^permeable GluR2) were correlated with decreased invasiveness 12. On the other hand, endogenous GluR2 was expressed in low-grade tumor specimens but not in high-grade tumor samples. Moreover, downregulation of GluR2 accelerated cell proliferation in glioma cells 19. In our study, both endogenous and exogenous knockdown of GluR2 decreased proliferation, migration and invasion of EC cells, which facilitated the potential role of GluR2 in targeted therapy of EC. In the detection of GluR2 in EC tissues using IHC, the expression of GluR2 is upregulated in EC and atypical hyperplasia (AH) endometrium compared to that in normal endometrium. Interestingly, the GluR2 expression was even higher in the AH group than in EC specimens. Thus, GluR2 activation might be crucial in the transformation of AH to cancer, or the precancerous EC cells might already be able to initiate PNI similar to the precancerous pancreatic cells reported previously 43. Although GluR2 antagonist revealed an anti-proliferative effect on EC cells, the Ishikawa cells were not affected by the agonist. However, on one hand, the agonist worked not only on GluR2 but also on GluR1 (another subtype of AMPARs). On the other hand, the result also suggested that GluR2 might be necessary but not sufficient for EC cell proliferation 43.

However, whether the cancer cells drive the outgrowth of nerves is not detected in the present study but rather in later on-going studies in our group. Furthermore, the nerve microenvironment itself is truly complicated; prior studies have reported that chemokine 43, neuropeptide 11, nerve growth factor 45 as well as glutamate 12 were correlated with nerve and cancer crosstalk. With demonstration that the concentration of glutamate was significantly elevated after coculture with DRG neurons, the extra elements in the coculture supernatant, which might contribute to metastasis of EC cells, were not detected. In addition to DRG neurons, Schwann cells could also migrate toward cancer cells before the onset of cancer invasion 46, and the impact of Schwann cells might also be included in the further investigation of nerve cancer interaction.

## Conclusion

In conclusion, AMPA receptor 2 (GluR2) was expressed in both EC tissue and cell lines, and DRG neurons facilitated metastasis of EC cells via GluR2, whereas the effect could be inhibited by both endogenous/exogenous knockdown of GluR2. The results revealed the potential role of GluR2 in the prediction of distant metastasis in EC, although the findings need to be confirmed by further studies. Moreover, the effects of emotions, nerves and endocrine on EC cells remain to be detected in the future.

## Supplementary Material

Supplementary table and information.

Supplementary figure S1.
