# Supplementary material for: DRG Neurons Promote Perineural Invasion of Endometrial Cancer via GluR2
Source: J Cancer. 2020 Feb 10;11(9):2518–28. doi: 10.7150/jca.40055 (PMC7066017; doi:10.7150/jca.40055)
Supplement: Supplementary file 1 — Supplementary table and information. [file jcav11p2518s1.pdf]

Supplementary Table 1. PCR Array results of nerve related genes in endometrial cancer (test) and para-cancer (control) tissues

Symbol	Well	AVG ΔC$_t$ (Ct(GOI) - Ave Ct (HKG))		2^-ΔC$_t$		Fold Change
		Test Sample	Control Sample	Test Sample	Control Sample	Test Sample /Control Sample
ADRA1A	1	28.67	27.96	2.3E-09	3.8E-09	0.61
ADRA1D	2	26.41	24.36	1.1E-08	4.6E-08	0.24
ADRA2A	3	21.27	21.44	4.0E-07	3.5E-07	1.12
ADRA1B	4	24.61	25.57	3.9E-08	2.0E-08	1.94
ADRA2B	5	28.14	26.29	3.4E-09	1.2E-08	0.28
ADRA2C	6	22.65	21.72	1.5E-07	2.9E-07	0.52
ADRB1	7	25.97	25.47	1.5E-08	2.2E-08	0.70
ADRB2	8	22.89	23.74	1.3E-07	7.1E-08	1.80
ADRB3	9	27.38	27.81	5.7E-09	4.2E-09	1.35
CHRM1	10	27.79	27.05	4.3E-09	7.2E-09	0.60
CHRM4	11	27.48	27.64	5.4E-09	4.8E-09	1.12
CHRM5	12	27.98	27.64	3.8E-09	4.8E-09	0.79
CHRNA3	13	25.94	23.82	1.6E-08	6.7E-08	0.23
CHRNA4	14	28.45	27.16	2.7E-09	6.7E-09	0.41
CHRNA5	15	24.26	23.75	5.0E-08	7.1E-08	0.70
CHRNA6	16	26.59	25.64	9.9E-09	1.9E-08	0.52
CHRNA7	17	25.60	25.74	2.0E-08	1.8E-08	1.10
CHRNE	18	26.63	25.42	9.7E-09	2.2E-08	0.43
DRD1	19	28.92	27.80	2.0E-09	4.3E-09	0.46
DRD2	20	24.01	25.84	5.9E-08	1.7E-08	3.54
DRD3	21	25.99	26.00	1.5E-08	1.5E-08	1.01
DRD4	22	23.28	22.51	9.8E-08	1.7E-07	0.59
DRD5	23	24.52	24.68	4.2E-08	3.7E-08	1.12
GABRA1	24	28.59	28.98	2.5E-09	1.9E-09	1.30
GABRA2	25	26.64	25.36	9.6E-09	2.3E-08	0.41
GABRA4	26	26.19	26.75	1.3E-08	8.9E-09	1.47
GABRA5	27	30.62	27.42	6.1E-10	5.6E-09	0.11

GABRA6	28	27.05	24.85	7.2E-09	3.3E-08	0.22
GABRB1	29	26.94	26.48	7.7E-09	1.1E-08	0.72
GABRB3	30	26.18	25.01	1.3E-08	3.0E-08	0.44
GABRD	31	30.80	29.32	5.3E-10	1.5E-09	0.36
GABRE	32	26.49	26.49	1.1E-08	1.1E-08	1.00
GABRG1	33	24.91	24.14	3.2E-08	5.4E-08	0.59
GABRG2	34	27.42	27.16	5.6E-09	6.7E-09	0.84
GABRG3	35	23.25	23.27	1.0E-07	9.9E-08	1.02
GABRQ	36	25.83	24.73	1.7E-08	3.6E-08	0.47
GABBR1	37	23.80	22.07	6.9E-08	2.3E-07	0.30
GABBR2	38	25.81	25.17	1.7E-08	2.7E-08	0.64
GABRR1	39	27.14	27.01	6.7E-09	7.4E-09	0.91
GABRR2	40	26.04	25.59	1.5E-08	2.0E-08	0.73
GRIA1	41	26.50	26.17	1.1E-08	1.3E-08	0.80
GRIA2	42	18.82	22.22	2.2E-06	2.0E-07	10.58
GRIA3	43	24.76	23.53	3.5E-08	8.3E-08	0.43
GRIK1	44	26.17	25.25	1.3E-08	2.5E-08	0.53
GRIK2	45	24.01	23.83	5.9E-08	6.7E-08	0.88
GRIK4	46	27.48	27.95	5.3E-09	3.9E-09	1.39
GRIK5	47	26.60	27.04	9.8E-09	7.2E-09	1.35
GRIN1	48	29.10	29.97	1.7E-09	9.5E-10	1.82
GRIN2A	49	31.07	31.19	4.4E-10	4.1E-10	1.09
GRIN2B	50	31.37	32.37	3.6E-10	1.8E-10	2.01
GRIN2C	51	27.05	27.95	7.2E-09	3.9E-09	1.86
GRM1	52	26.11	26.42	1.4E-08	1.1E-08	1.24
GRM3	53	28.36	29.49	2.9E-09	1.3E-09	2.19
GRM4	54	26.85	26.78	8.2E-09	8.7E-09	0.95
GRM5	55	26.12	27.03	1.4E-08	7.3E-09	1.89
GRM6	56	27.62	28.14	4.9E-09	3.4E-09	1.44
GRM7	57	25.84	24.51	1.7E-08	4.2E-08	0.40
GRM8	58	27.63	26.55	4.8E-09	1.0E-08	0.47
HTR1A	59	27.32	28.20	6.0E-09	3.3E-09	1.83
HTR1B	60	28.43	28.36	2.8E-09	2.9E-09	0.95
HTR1D	61	26.65	26.16	9.5E-09	1.3E-08	0.71

HTR1F	62	23.21	25.06	1.0E-07	2.9E-08	3.61
HTR2A	63	25.79	25.91	1.7E-08	1.6E-08	1.09
HTR2C	64	25.81	26.21	1.7E-08	1.3E-08	1.31
HTR3A	65	26.00	25.82	1.5E-08	1.7E-08	0.88
HTR4	66	26.59	26.13	9.9E-09	1.4E-08	0.73
HTR7	67	25.76	25.83	1.8E-08	1.7E-08	1.05
HTR1E	68	25.24	23.80	2.5E-08	6.9E-08	0.37
HTR2B	69	21.10	19.67	4.5E-07	1.2E-06	0.37
HTR3B	70	27.28	27.54	6.1E-09	5.1E-09	1.20
HTR3C	71	28.20	27.87	3.2E-09	4.1E-09	0.79
HTR3D	72	27.67	27.49	4.7E-09	5.3E-09	0.88
HTR3E	73	29.47	26.81	1.3E-09	8.5E-09	0.16
HTR5A	74	27.16	26.83	6.7E-09	8.4E-09	0.80
HTR6	75	27.77	27.36	4.4E-09	5.8E-09	0.75
NEFH	76	25.24	22.57	2.5E-08	1.6E-07	0.16
NEFL	77	25.93	25.30	1.6E-08	2.4E-08	0.65
NEFM	78	24.85	22.13	3.3E-08	2.2E-07	0.15
AVPR1A	79	23.12	21.71	1.1E-07	2.9E-07	0.37
AVPR1B	80	29.19	29.22	1.6E-09	1.6E-09	1.02
HRH1	81	22.23	20.60	2.0E-07	6.3E-07	0.32
HRH4	82	27.09	26.87	7.0E-09	8.1E-09	0.86
NPY2R	83	27.11	27.97	6.9E-09	3.8E-09	1.81
NPY5R	84	25.60	26.21	2.0E-08	1.3E-08	1.52
SSTR1	85	24.35	26.34	4.7E-08	1.2E-08	3.97
SSTR2	86	26.02	26.25	1.5E-08	1.3E-08	1.18
SSTR4	87	28.64	31.56	2.4E-09	3.2E-10	7.54
TACR1	88	25.80	27.18	1.7E-08	6.6E-09	2.60
TACR2	89	26.99	26.76	7.5E-09	8.8E-09	0.85
TACR3	90	26.91	27.20	7.9E-09	6.5E-09	1.22
BRS3	91	27.86	27.97	4.1E-09	3.8E-09	1.08
CCKBR	92	31.21	29.66	4.0E-10	1.2E-09	0.34
CNR1	93	28.00	27.99	3.7E-09	3.8E-09	0.99
GCGR	94	32.75	31.57	1.4E-10	3.1E-10	0.44
GRPR	95	27.19	27.18	6.5E-09	6.6E-09	1.00

HCRTR2	96	29.52	28.38	1.3E-09	2.9E-09	0.45
NTSR2	97	28.01	27.31	3.7E-09	6.0E-09	0.62
OXTR	98	28.52	29.69	2.6E-09	1.2E-09	2.26
PROKR2	99	29.52	28.77	1.3E-09	2.2E-09	0.60
SCTR	100	30.87	32.55	5.1E-10	1.6E-10	3.22
TSPO	101	21.59	20.54	3.2E-07	6.6E-07	0.48
COMT	102	27.26	26.01	6.2E-09	1.5E-08	0.42
DBH	103	28.59	27.98	2.5E-09	3.8E-09	0.65
DDC	104	24.66	26.63	3.8E-08	9.6E-09	3.91
EPHB1	105	27.46	26.56	5.4E-09	1.0E-08	0.53
GDNF	106	26.93	26.46	7.8E-09	1.1E-08	0.72
GFAP	107	28.25	28.79	3.1E-09	2.2E-09	1.45
MAOA	108	21.09	21.09	4.5E-07	4.5E-07	1.00
MAOB	109	19.81	19.31	1.1E-06	1.5E-06	0.70
NR4A1	110	20.55	22.83	6.5E-07	1.3E-07	4.85
NR4A3	111	25.00	26.78	3.0E-08	8.7E-09	3.45
PDYN	112	28.98	29.31	1.9E-09	1.5E-09	1.26
PTGS2	113	25.28	24.69	2.5E-08	3.7E-08	0.67
SYN2	114	27.10	26.34	6.9E-09	1.2E-08	0.59
SLC18A1	115	28.83	28.33	2.1E-09	3.0E-09	0.71
SLC18A2	116	22.20	20.62	2.1E-07	6.2E-07	0.33
SLC6A3	117	27.92	26.80	3.9E-09	8.6E-09	0.46
SLC6A4	118	31.28	30.53	3.8E-10	6.4E-10	0.60
TDO2	119	24.99	23.99	3.0E-08	6.0E-08	0.50
TH	120	32.38	29.90	1.8E-10	1.0E-09	0.18
TPH1	121	26.86	27.48	8.2E-09	5.4E-09	1.54
TPH2	122	26.82	26.88	8.5E-09	8.1E-09	1.05
CHAT	123	29.38	29.00	1.4E-09	1.9E-09	0.77

GENETIC TESTING BIOTECHNOLOGY

Cell Line Authentication Service

STR Profile Report

**Sample Submitted By:**	Dr. Yudong Wang<br>International Peace Maternity and Child Health Hospital, Shanghai Jiao Tong University School of Medicine
**Email Address:**	niting6688@126.com
**Sales Order:**	180124C
**Cell Line Designation:**	HEC-1-A
**Date Sample Received:**	Jan 24th, 2018
**Report Date:**	Jan 25th, 2018

Methodology: Nineteen short tandem repeat (STR) loci plus the gender determining locus, Amelogenin, were amplified using the commercially available EX20 Kit from AGCU. The cell line sample was processed using the ABI Prism® 3500 Genetic Analyzer. Data were analyzed using GeneMapper® ID-X v1.4 software (Applied Biosystems). Appropriate positive and negative controls were run and confirmed for each sample submitted.

Data Interpretation: Cell lines were authenticated using Short Tandem Repeat (STR) analysis as described in 2012 in ANSI Standard (ASN-0002) by the ATCC Standards Development Organization (SDO) and in Capes-Davis et al., Match criteria for human cell line authentication: Where do we draw the line? Int J Cancer. 2013;132(11):2510-9.

GTB™ performs STR Profiling following ISO 9001:2008 and ISO/IEC 17025:2005 quality standards.
There are no warranties with respect to the services or results supplied, express or implied, including, without limitation, any implied warranty of merchantability or fitness for a particular purpose. Genetic Testing Biotechnology (GTB) is not liable for any damages or injuries resulting from receipt and/or improper, inappropriate, negligent or other wrongful use of the test results supplied, and/or from misidentification, misrepresentation, or lack of accuracy of those results. Your exclusive remedy against GTB and those supplying materials used in the services for any losses or damage of any kind whatsoever, whether in contract, tort, or otherwise, shall be, at GTB's option, refund of the fee paid for such service or repeat of the service.

The GTB™ is a registered trademark of Genetic Testing Biotechnology Corporation (Suzhou).

Technical Questions?
GTB Technical Support
+86-512-62806339
service@jsdna.org
Section 303, Yixin BLD
SIP, Suzhou, 215123
Jiangsu, P.R. China

Ordering Questions?
order@jsdna.org
GTB Corporation
+86-512-62806339
Section 303, Yixin BLD
SIP, Suzhou, 215123
Jiangsu, P.R. China

GENETIC TESTING BIOTECHNOLOGY

Cell Line Authentication Service

STR Profile Report

Sales Order: 180124C

	**Test Results for Submitted Sample**				**DSMZ Reference Database Profile**	
**Loci**	**Query Profile:** HEC-1-A				**Database Profile:** HEC-1-A	
Amelogenin	X				X	
D3S1358	15					
D13S317	*11*				11	12
D7S820	9	11			9	11
D16S539	*12*				12	13
Penta E	11					
TPOX	8	11			8	11
TH01	6	7			6	7
D2S1338	18	19				
CSF1PO	10	12			10	12
Penta D	9	13				
D19S433	13					
vWA	18	19			18	19
D21S11	30	31				
D18S51	16	17	21	22		
D6S1043	12	18				
D8S1179	13	14				
D5S818	11	15			11	15
D12S391	19					
FGA	21	22				

The allele match algorithm compares the 8 core loci plus amelogenin only, even though alleles from all loci will be reported when available.

Note: Loci highlighted in grey (8 core STR loci plus Amelogenin) can be made public to verify cell identity. In order to protect the identity of the donor, ***please do not publish*** *the allele calls from all the STR loci tested.*

Explanation of Test Results

Cell lines with ≥80% match are considered to be related; i.e., derived from a common ancestry. Cell lines with between a 55% to 80% match require further profiling for authentication of relatedness.

- [ ] The submitted sample profile is human, but not a match for any profile in the DSMZ STR database.
- [ ] The submitted profile is an exact match for the following human cell line(s) in the DSMZ STR database (8 core loci plus Amelogenin):
- [x] The submitted profile is similar to the following DSMZ human cell line(s): HEC-1-A (89% match)

e-Signature, Technician:

Digitally signed by Faye Wong
DN: cn=Faye Wong, o=Genetic Testing Biotechnology (Suzhou), ou=DNA Typing Section, email=order@jsdna.org, c=CN
Date: 2018.01.25 16:29:58 +08'00'

GENETIC TESTING BIOTECHNOLOGY
GUARANTEE
CELL LINE AUTHENTICATION

e-Signature, Reviewer:

Digitally signed by Alan Cui
DN: cn=Alan Cui, o=Genetic Testing Biotechnology (Suzhou), ou=Supervision Section, email=service@jsdna.org, c=CN
Date: 2018.01.25 16:31:30 +08'00'

More information

Addendum: Electropherogram/matching results for the customer's sample set 1 of 1

GENETIC TESTING BIOTECHNOLOGY

Cell Line Authentication Service

STR Profile Report

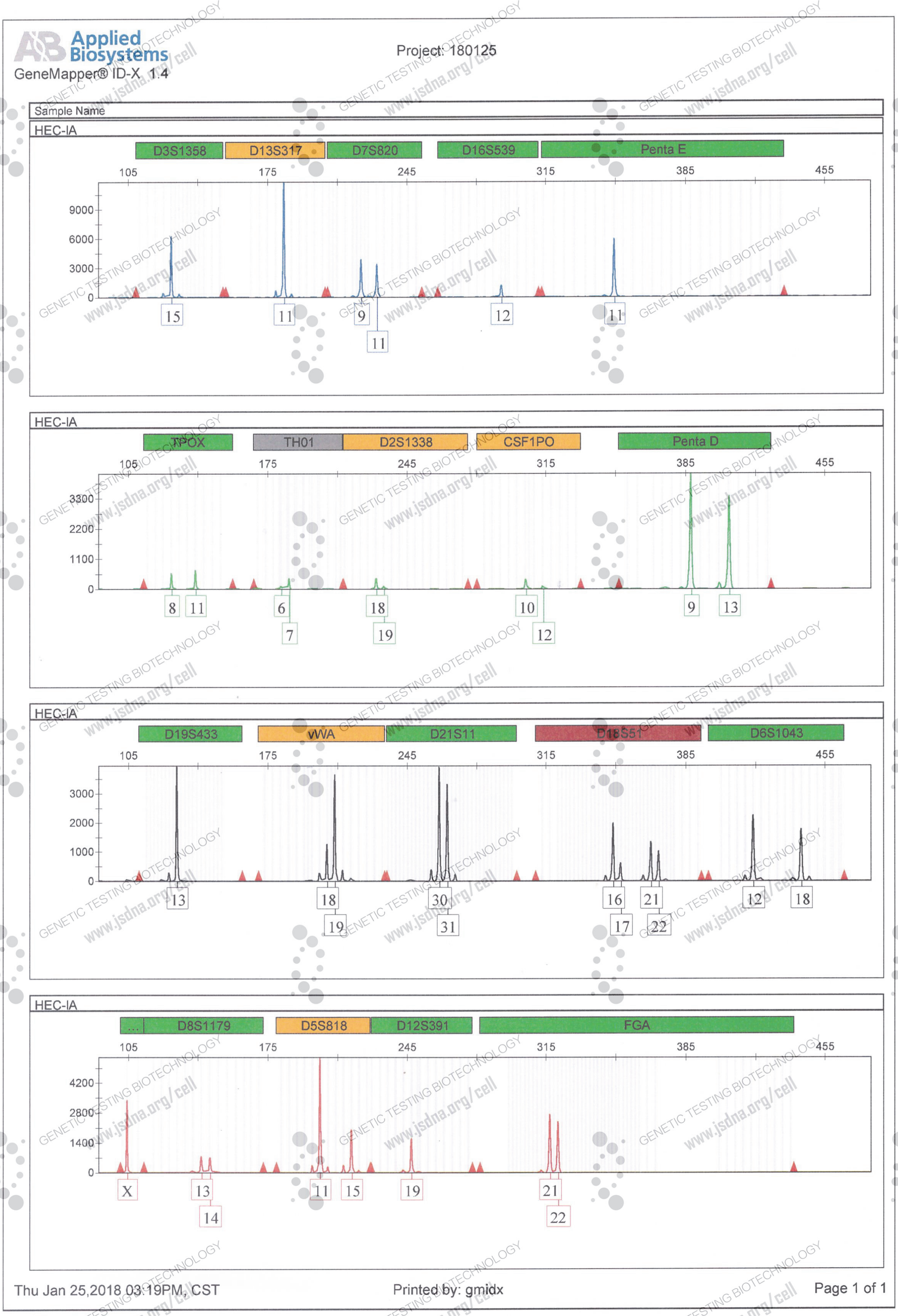

GENETIC TESTING BIOTECHNOLOGY www.jsdna.org/cell

Cell Line Authentication Service
STR Profile Report

Sample Submitted By: Dr. Yudong Wang
International Peace Maternity and Child Health Hospital, Shanghai Jiao Tong University School of Medicine

Email Address: niting6688@126.com

Sales Order: 180124B

Cell Line Designation: ISHIKAWA

Date Sample Received: Jan 24th, 2018

Report Date: Jan 25th, 2018

Methodology: Nineteen short tandem repeat (STR) loci plus the gender determining locus, Amelogenin, were amplified using the commercially available EX20 Kit from AGCU. The cell line sample was processed using the ABI Prism® 3500 Genetic Analyzer. Data were analyzed using GeneMapper® ID-X v1.4 software (Applied Biosystems). Appropriate positive and negative controls were run and confirmed for each sample submitted.

Data Interpretation: Cell lines were authenticated using Short Tandem Repeat (STR) analysis as described in 2012 in ANSI Standard (ASN-0002) by the ATCC Standards Development Organization (SDO) and in Capes-Davis et al., Match criteria for human cell line authentication: Where do we draw the line? Int J Cancer. 2013;132(11):2510-9.

GTB™ performs STR Profiling following ISO 9001:2008 and ISO/IEC 17025:2005 quality standards.

There are no warranties with respect to the services or results supplied, express or implied, including, without limitation, any implied warranty of merchantability or fitness for a particular purpose. Genetic Testing Biotechnology (GTB) is not liable for any damages or injuries resulting from receipt and/or improper, inappropriate, negligent or other wrongful use of the test results supplied, and/or from misidentification, misrepresentation, or lack of accuracy of those results. Your exclusive remedy against GTB and those supplying materials used in the services for any losses or damage of any kind whatsoever, whether in contract, tort, or otherwise, shall be, at GTB's option, refund of the fee paid for such service or repeat of the service.

The GTB™ is a registered trademark of Genetic Testing Biotechnology Corporation (Suzhou).

Technical Questions?
GTB Technical Support
+86-512-62806339
service@jsdna.org
Section 303, Yixin BLD
SIP, Suzhou, 215123
Jiangsu, P.R. China

Ordering Questions?
order@jsdna.org
GTB Corporation
+86-512-62806339
Section 303, Yixin BLD
SIP, Suzhou, 215123
Jiangsu, P.R. China

GENETIC TESTING BIOTECHNOLOGY

Cell Line Authentication Service

STR Profile Report

Sales Order: 180124B

Test Results for Submitted Sample				DSMZ Reference Database Profile	
**Loci**	**Query Profile:** ISHIKAWA			**Database Profile:** ISHIKAWA	
Amelogenin	X			X	
D3S1358	15	16			
D13S317	9	12		9	12
D7S820	9	10		9	10
D16S539	9			9	
Penta E	11	19	21		
TPOX	8			8	
TH01	9	10		9	10
D2S1338	19	21			
CSF1PO	11	12		11	12
Penta D	10	11			
D19S433	12.2	14	15		
vWA	14	17		14	17
D21S11	28				
D18S51	12	20	21		
D6S1043	12	18			
D8S1179	13	16			
D5S818	10	11		10	11
D12S391	18	20	21		
FGA	21	22			

The allele match algorithm compares the 8 core loci plus amelogenin only, even though alleles from all loci will be reported when available.

Note: Loci highlighted in grey (8 core STR loci plus Amelogenin) can be made public to verify cell identity. In order to protect the identity of the donor, ***please do not publish*** *the allele calls from all the STR loci tested.*

Explanation of Test Results

Cell lines with ≥80% match are considered to be related; i.e., derived from a common ancestry. Cell lines with between a 55% to 80% match require further profiling for authentication of relatedness.

- ☐ The submitted sample profile is human, but not a match for any profile in the DSMZ STR database.
- ☑ The submitted profile is an exact match for the following human cell line(s) in the DSMZ STR database (8 core loci plus Amelogenin): ISHIKAWA
- ☐ The submitted profile is similar to the following DSMZ human cell line(s):

e-Signature, Technician:

Digitally signed by Faye Wong
DN: cn=Faye Wong, o=Genetic Testing Biotechnology (Suzhou), ou=DNA Typing Section, email=order@jsdna.org, c=CN
Date: 2018.01.25 16:29:34 +08'00'

GENETIC TESTING BIOTECHNOLOGY — GUARANTEE — CELL LINE AUTHENTICATION

e-Signature, Reviewer:

Digitally signed by Alan Cui
DN: cn=Alan Cui, o=Genetic Testing Biotechnology (Suzhou), ou=Supervision Section, email=service@jsdna.org, c=CN
Date: 2018.01.25 16:31:07 +08'00'

More information

Addendum: Electropherogram/matching results for the customer's sample set 1 of 1

GENETIC TESTING BIOTECHNOLOGY

Cell Line Authentication Service

STR Profile Report

Applied Biosystems
GeneMapper® ID-X 1.4

Project: 180125

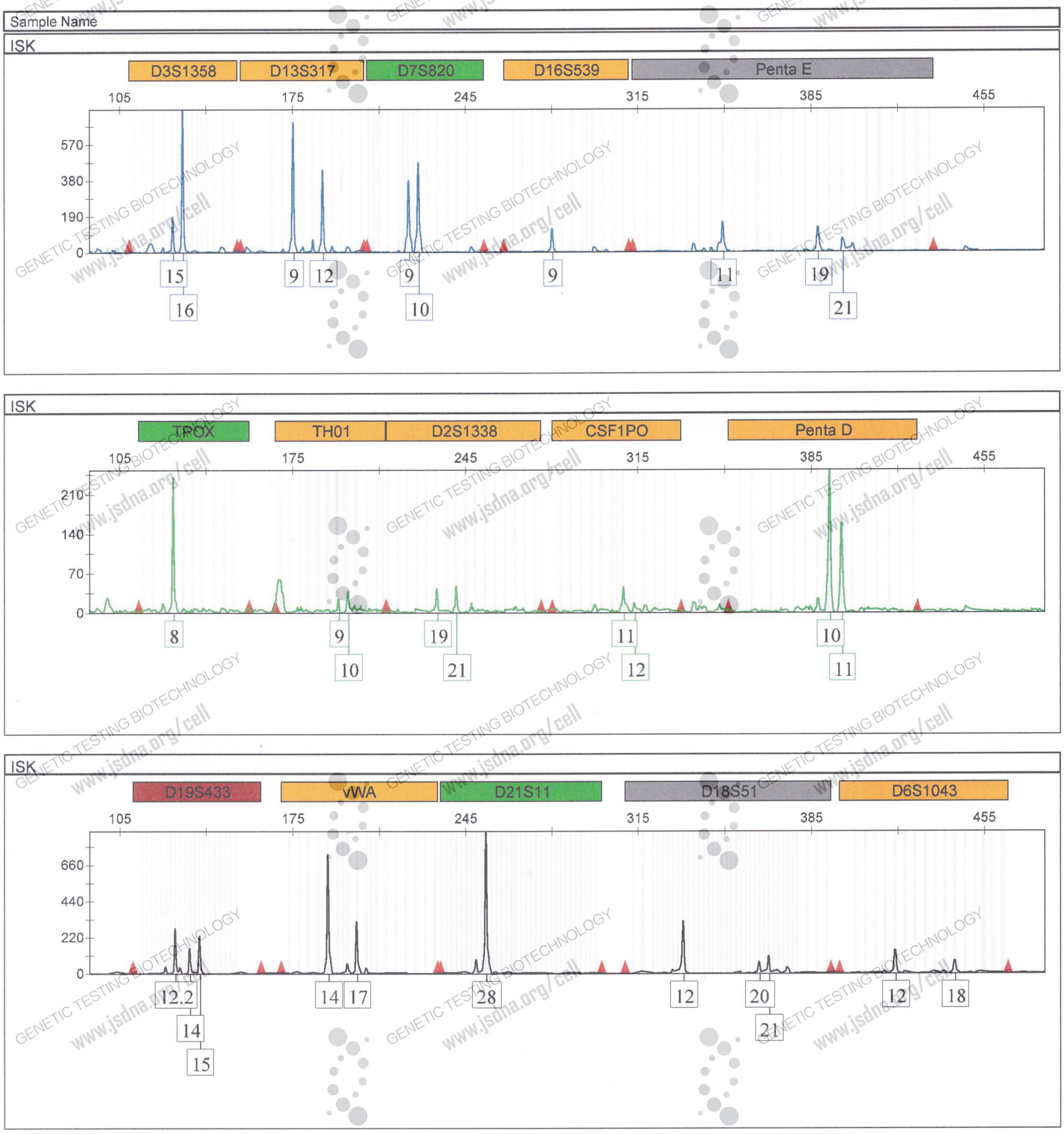

Thu Jan 25,2018 03:19PM, CST

Printed by: gmidx

Page 1 of 2

GENETIC TESTING BIOTECHNOLOGY www.jsdna.org/cell

GENETIC TESTING BIOTECHNOLOGY

Cell Line Authentication Service

STR Profile Report

Applied Biosystems
GeneMapper® ID-X 1.4

Project: 180125

Sample Name

ISK

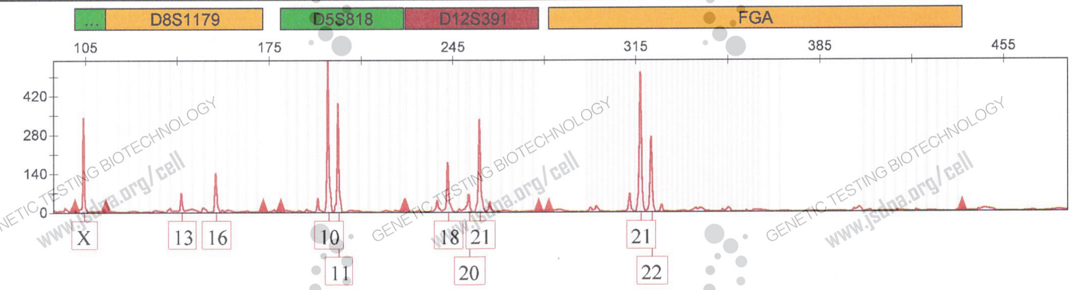

Thu Jan 25,2018 03:19PM, CST

Printed by: gmidx

Page 2 of 2

GENETIC TESTING BIOTECHNOLOGY www.jsdna.org/cell

GENETIC TESTING BIOTECHNOLOGY

Cell Line Authentication Service

STR Profile Report

Sample Submitted By: Dr. Yudong Wang
International Peace Maternity and Child Health Hospital, Shanghai Jiao Tong University School of Medicine

Email Address: niting6688@126.com

Sales Order: 180211A

Cell Line Designation: KLE

Date Sample Received: Feb 11th, 2018

Report Date: Feb 12th, 2018

Methodology: Nineteen short tandem repeat (STR) loci plus the gender determining locus, Amelogenin, were amplified using the commercially available EX20 Kit from AGCU. The cell line sample was processed using the ABI Prism® 3500 Genetic Analyzer. Data were analyzed using GeneMapper® ID-X v1.4 software (Applied Biosystems). Appropriate positive and negative controls were run and confirmed for each sample submitted.

Data Interpretation: Cell lines were authenticated using Short Tandem Repeat (STR) analysis as described in 2012 in ANSI Standard (ASN-0002) by the ATCC Standards Development Organization (SDO) and in Capes-Davis et al., Match criteria for human cell line authentication: Where do we draw the line? Int J Cancer. 2013;132(11):2510-9.

GTB™ performs STR Profiling following ISO 9001:2008 and ISO/IEC 17025:2005 quality standards.

There are no warranties with respect to the services or results supplied, express or implied, including, without limitation, any implied warranty of merchantability or fitness for a particular purpose. Genetic Testing Biotechnology (GTB) is not liable for any damages or injuries resulting from receipt and/or improper, inappropriate, negligent or other wrongful use of the test results supplied, and/or from misidentification, misrepresentation, or lack of accuracy of those results. Your exclusive remedy against GTB and those supplying materials used in the services for any losses or damage of any kind whatsoever, whether in contract, tort, or otherwise, shall be, at GTB's option, refund of the fee paid for such service or repeat of the service.

The GTB™ is a registered trademark of Genetic Testing Biotechnology Corporation (Suzhou).

Technical Questions?
GTB Technical Support
+86-512-62806339
service@jsdna.org
Section 303, Yixin BLD
SIP, Suzhou, 215123
Jiangsu, P.R. China

Ordering Questions?
order@jsdna.org
GTB Corporation
+86-512-62806339
Section 303, Yixin BLD
SIP, Suzhou, 215123
Jiangsu, P.R. China

GENETIC TESTING BIOTECHNOLOGY

Cell Line Authentication Service

STR Profile Report

Sales Order: 180211A

Test Results for Submitted Sample			DSMZ Reference Database Profile	
**Loci**	**Query Profile:** KLE		**Database Profile:** KLE	
Amelogenin	X		X	
D3S1358	17			
D13S317	12		12	
D7S820	11	12	11	12
D16S539	11	12	11	12
Penta E	7			
TPOX	8	11	8	11
TH01	6	7	6	7
D2S1338	18	19		
CSF1PO	13	14	13	14
Penta D	13			
D19S433	15			
vWA	16		16	
D21S11	28	30		
D18S51	13	17		
D6S1043	13	14		
D8S1179	8	14		
D5S818	9	12	9	12
D12S391	20	25		
FGA	23	25		

The allele match algorithm compares the 8 core loci plus amelogenin only, even though alleles from all loci will be reported when available.

Note: Loci highlighted in grey (8 core STR loci plus Amelogenin) can be made public to verify cell identity. In order to protect the identity of the donor, ***please do not publish*** *the allele calls from all the STR loci tested.*

Explanation of Test Results

Cell lines with ≥80% match are considered to be related; i.e., derived from a common ancestry. Cell lines with between a 55% to 80% match require further profiling for authentication of relatedness.

☐ The submitted sample profile is human, but not a match for any profile in the DSMZ STR database.

☑ The submitted profile is an exact match for the following human cell line(s) in the DSMZ STR database (8 core loci plus Amelogenin): KLE

☐ The submitted profile is similar to the following DSMZ human cell line(s):

e-Signature, Technician:

Digitally signed by Faye Wong
DN: cn=Faye Wong, o=Genetic Testing Biotechnology (Suzhou), ou=DNA Typing Section, email=order@jsdna.org, c=CN
Date: 2018.02.12 13:36:27 +08'00'

e-Signature, Reviewer:

Digitally signed by Alan Cui
DN: cn=Alan Cui, o=Genetic Testing Biotechnology (Suzhou), ou=Supervision Section, email=service@jsdna.org, c=CN
Date: 2018.02.12 13:36:45 +08'00'

More information

Addendum: Electropherogram/matching results for the customer's sample set 1 of 1

GENETIC TESTING BIOTECHNOLOGY

Cell Line Authentication Service

STR Profile Report

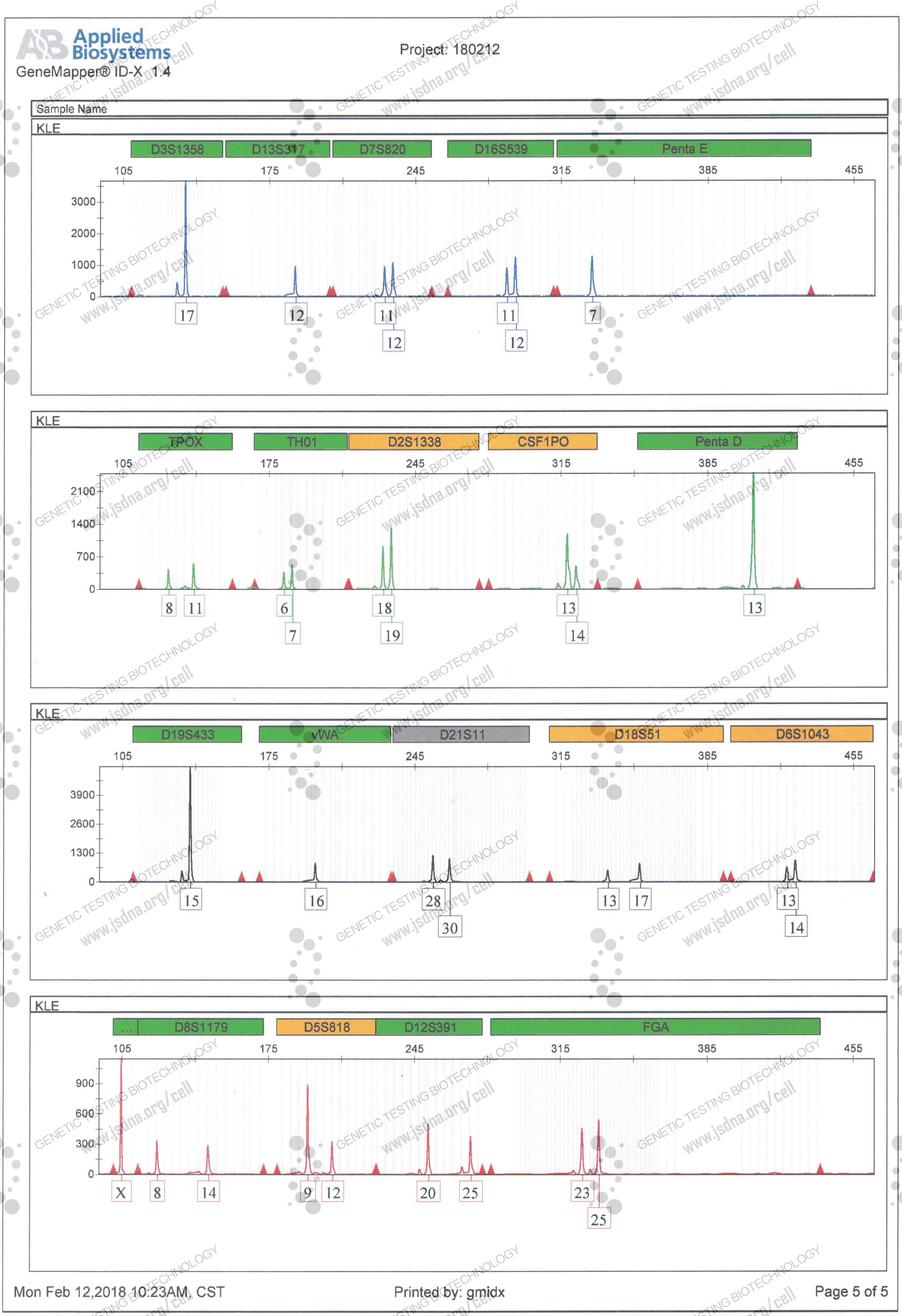

GENETIC TESTING BIOTECHNOLOGY www.jsdna.org/cell
