# Supplementary material for: DRG Neurons Promote Perineural Invasion of Endometrial Cancer via GluR2
Source: J Cancer. 2020 Feb 10;11(9):2518–28. doi: 10.7150/jca.40055 (PMC7066017; doi:10.7150/jca.40055)

Figure S1. Screening of cell lines prone to PNI. (A) The other two EC cell lines HEC-1A and KLE were also cocultured with DRG at day 2. The interaction of nerve and cancer cells were not observed (50× magnification). (B) PNI in cervical cancer cell line Hela as positive control. Pan-cytokeratin (pan-CK), green (100× magnification), scale bar, 50μm.

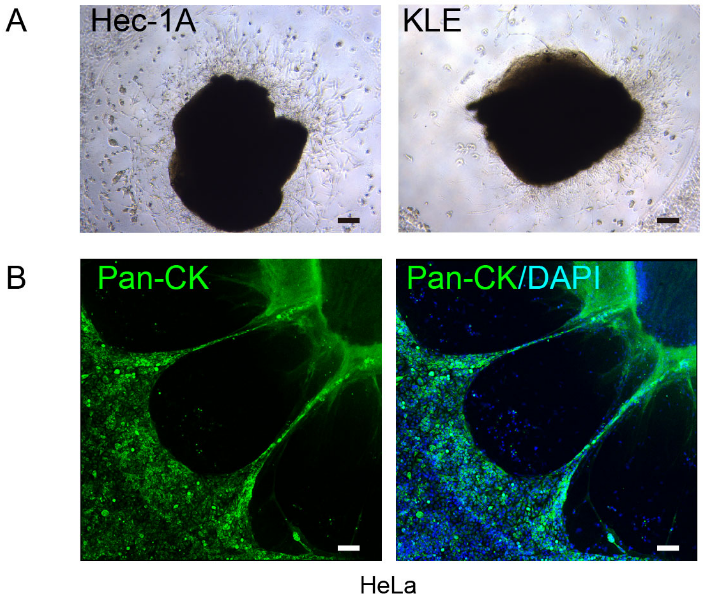

Supplement: Supplementary file 2 — Supplementary figure S1. [file jcav11p2518s2.pdf]
